# Linkage of Bacterial Protein Synthesis and Presentation of MHC Class I-Restricted *Listeria monocytogenes*-Derived Antigenic Peptides

**DOI:** 10.1371/journal.pone.0033335

**Published:** 2012-03-12

**Authors:** Silke Grauling-Halama, Simone Schenk, Andreas Bubert, Gernot Geginat

**Affiliations:** 1 Institut für medizinische Mikrobiologie und Hygiene, Fakultät für Medizin Mannheim der Universität Heidelberg, Universität Heidelberg, Mannheim, Germany; 2 Merck KG, Darmstadt, Germany; 3 Institut für Medizinische Mikrobiologie, Universitätsklinikum Magdeburg, Magdeburg, Germany; Kyushu Institute of Technology, Japan

## Abstract

The processing and MHC class I-restricted presentation of antigenic peptides derived from the p60 protein of the facultative intracellular bacterium *Listeria monocytogenes* is tightly linked to bacterial protein synthesis. We used non-linear regression analysis to fit a mathematical model of bacterial antigen processing to a published experimental data set showing the accumulation and decay of p60-derived antigenic peptides in *L. monocytogenes*-infected cells. Two alternative models equally describe the experimental data. The simulation accounting for a stable and a hypothetical rapidly degraded form of antigen predicts that the antigenic peptides p60 217–225 and p60 449–457 are derived from a putative instable form of p60 with an average intracellular half-life of approximately 3 minutes accounting for approximately 31% of all p60 molecules synthesized. The alternative model predicts that both antigenic peptides are processed from p60 degraded intracellularly with a half-life of 109 min and that antigen processing only occurs as long as bacterial protein synthesis is not inhibited. In order to decide between both models the intracellular accumulation of p60 in infected cells was studied experimentally and compared with model predictions. Inhibition of p60 degradation by the proteasome inhibitor epoxomicin revealed that during the first 3 h post infection approximately 30% of synthesized p60 molecules were degraded. This value is significantly lower than the approximately 50% degradation of p60 that would be expected in the presence of the predicted putative short-lived state of p60 and also fits precisely with the predictions of the alternative model, indicating that the tight connection of bacterial protein biosynthesis and antigen processing and presentation of *L. monocyctogenes*-derived antigenic peptides is not caused by the presence of a highly instable antigenic substrate.

## Introduction

The immune surveillance by cytotoxic CD8 T cells of cells infected by intracellular pathogens depends on the rapid presentation of pathogen-derived antigenic peptides in the context of MHC class I molecules on the surface of infected cells. The proteasome degrades pathogen-derived proteins and generates antigenic peptides that subsequently are transported into the endoplasmatic reticulum were binding to nascent MHC class I molecules occurs [1]. In eukaryotic cells proteins are degraded with largely different kinetics. Remarkably, also individual proteins are degraded intracellularly with different half-lifes. Approximately 30% of a newly synthesized protein enters the pool of rapidly degraded proteins (RDP) which are degraded in less than 10 min. The remainder protein enters the pool of slowly degraded proteins which exhibit a much longer half-life [2], [3], [4], [5].

A number of studies performed with virally infected cells suggest that RDP provide the majority of antigenic peptides that are transported by the transporters associated with antigen presentation (TAP) and presented in the context of MHC class I molecules on the surface of infected cells [5], [6], [7]. As RDP are rapidly degraded they don't accumulate intracellularly and thus blockade of protein synthesis would rapidly deprive their intracellular pool. Therefore the tight linkage of protein synthesis with antigen presentation can be interpreted as experimental evidence for the dependency of antigen presentation on RDP [8], [9]. It was proposed that rapid degradation of a protein is caused by errors in transcription, translation or folding. For these rapidly degraded proteins the term defective ribosomal products was introduced [10].

Studies by the Pamer laboratory revealed that the processing of two antigenic peptides, p60 217–225 and p60 449–457 derived from the p60 protein of the facultative intracellular bacterium *Listeria monocytogenes* is tightly connected to de novo p60 protein synthesis [11], [12]. During infection of cells with *L. monocytogenes* p60 accumulates and is intracellularly degraded with a rather slow half-life of approximately 90 min. Nevertheless, inhibition of bacterial protein synthesis promptly abrogates antigen processing.

In contrast to viral pathogens, bacteria provide their own protein biosynthesis apparatus. It is not known if in analogy to cellular RDP inside cells also a fraction of bacterial proteins is rapidly degraded.

In the current study the accumulation and decay of p60 in *L. monocytogenes*-infected cells was studied in order to decide whether putative p60-RDP are a major substrate for the processing of p60-derived antigenic peptides. Mathematical modelling of p60 accumulation and degradation predicted that approximately 31% of synthesized p60 is rapidly degraded with a short half-life of less than 5 min. These putative p60-RDP, however, were not detected experimentally, indicating that the tight linkage of bacterial antigen presentation is not caused by the presence of an intracellular pool of highly instable p60 molecules.

## Results

### Modelling of p60 antigen processing predicts an instable form of p60 as antigenic substrate

We used mathematical modelling in order to infer the intracellular half-life and the relative amount of the putative antigenic substrates from which p60 217–225 and p60 449–457 are processed from published experimental data. The data set used was published by Sijts and Pamer [11] and shows the accumulation of antigenic peptides in *L. monocytogenes*-infected cells as well as the subsequent decay of accumulated peptide/MHC complexes after inhibition of bacterial protein synthesis by tetracycline added at 4h after infection of cells.

The processing of p60-derived antigenic peptides was either simulated with hypothetical p60-RPD in addition to a slowly degraded form of p60, or alternatively only with a single form of p60.

In order to simulate the synthesis and the decay of putative p60-RDP the previously described antigen processing and presentation model [13] was expanded to represent two different states of an antigenic substrate which decay with individual intracellular half-life. Curve fitting to experimental data was performed by non-linear step-wise regression analysis with the following parameters: initial number o bacteria per cell: 1.25, bacterial doubling time: 60 min, bacterial p60 synthesis rate: 58 molecules/min/bacterium, p60 217–225 processing efficacy 1 peptide per 35 degraded p60 molecules, p60 217–225 half-life: 300 min. p60 449–457 processing efficacy 1 peptide per 1.7 degraded p60 molecules, p60 449–457 half-life: 60 min [11], [13].

**Figure 1:**
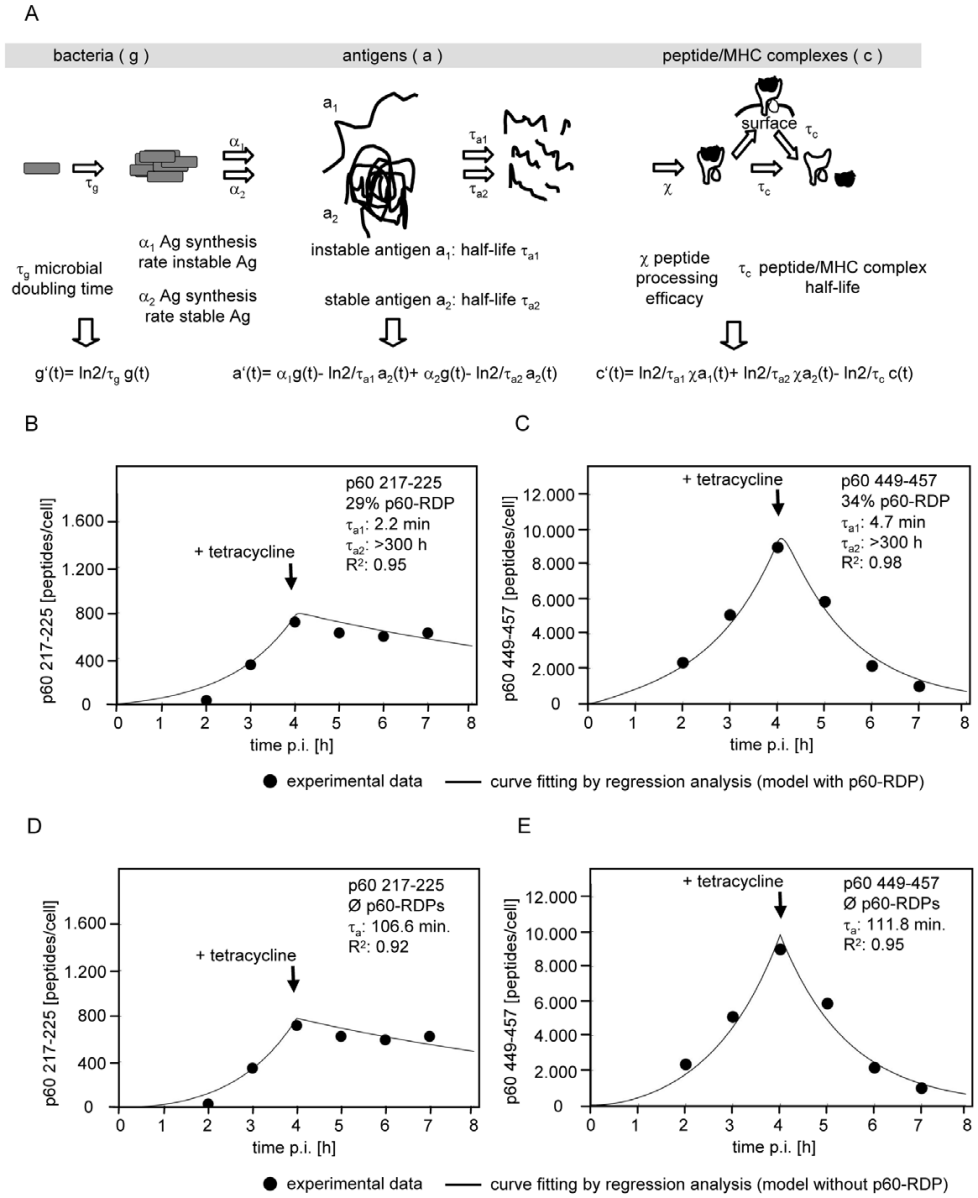
Mathematical modelling of p60 antigen processing. A. The interdependency of bacterial replication, antigen synthesis, antigen decay, processing of antigenic peptides, and the decay of peptide/MHC complexes is represented as a system of ordinary differential equations. The model assumes the existence of two states of an antigen that are synthesized with individual rates α_1_ and α_2_ and decay with different intracellular half-life τ_a1_ and τ_a2_. The differential equations were solved for distinct physiological scenarios defined by the bacterial doubling time τ_g_, the antigen synthesis rate α, and the antigen half-life τ_a_ (see supporting information: appendix S1). B–E. In order to model infected cells that were treated with tetracycline after 4 h of active bacterial replication the appropriate equations for t<4 h and t>4 h were merged to yield a stepwise function. This stepwise function was fitted to experimental data by non-linear regression analysis. Experimental data (filled circles) of the numbers of p60_217–225_ (B, D) and p60_449–457_ (C, E) peptides per cell at different time points after infection of cells with *L. monocytogenes* were taken from Sijts and Pamer 1997 [11]. In the experimental protocol after an initial period of 4 h of unrestricted bacterial replication and protein synthesis protein translation was inhibited by addition of tetracycline. Curve fitting was either performed on the basis of the above model assuming the presence of p60-RDP and assuming that previously accumulated p60 molecules are further processed and presented after inhibition of bacterial protein synthesis by tetracycline (B, C). Alternatively curve fitting was performed with a model without p60-RDP assuming that after inhibition of bacterial protein synthesis by tetracycline further antigen processing and presentation is blocked (D, E). Indicated are the results of curve fitting: the percentage of short-lived p60 molecules (p60-RDP) and the half-lifes of short-lived p60-RDP (τ_a1_) and long-lived p60 (τ_a2_), and the coefficient of determination (R^2^) of the curve fitting.

Fitting of the model to the p60 217–225 data set predicted an instable antigenic substrate (half-life 2.2 min) accounting for 28.8% of the total amount of p60 synthesized and a highly stable form of p60 with a half-life of >300 h (Fig. 1B). Fitting of the p60 449–457 data set predicted an instable antigenic substrate (half-life 4.7 min) accounting for 34.2% of the total amount of p60 synthesized and a highly stable form of p60 with a half-life of >300 h (Fig. 1C). The coefficients of determination (R^2^) for the p60 217–225 and p60 449–457 datasets were 0.95 and 0.98, respectively.

The above model accounts the p60 449–457 processing efficacy with one peptide per 1.7 p60 molecules degraded [13]. If the model is fitted with the maximum theoretical processing efficacy of one p60 449–457 peptide per one degraded p60 molecule a p60-RDP rate of 20.1% is predicted.

Alternatively, curve fitting was performed without p60-RDP (only a single form of antigenic substrate) but assuming that after inhibition of bacterial protein synthesis by tetracycline further antigen processing and presentation is blocked. For the p60 217–225 (Fig. 1D) and the p60 449–457 (Fig. 1E) data sets the model predicts a p60 half-life of 106.6 min (R^2^ = 0.92) and 111.8 min (R^2^ = 0.95), respectively.

Thus, taken together, both models quite precisely (R^2^>0.9) simulate the experimental datasets. The first model assuming the presence of p60-RDP predicts an average of 31.5% p60-RDP with a mean half-life of 3.4 min, whereas the alternative model without p60-RDP assuming the blockade of antigen processing and presentation after inhibition of bacterial protein synthesis predicts an average p60 half-life of 109 min.

### Quantitative analysis of p60 degradation in *L. monocytogenes*-infected cells

**Figure 2:**
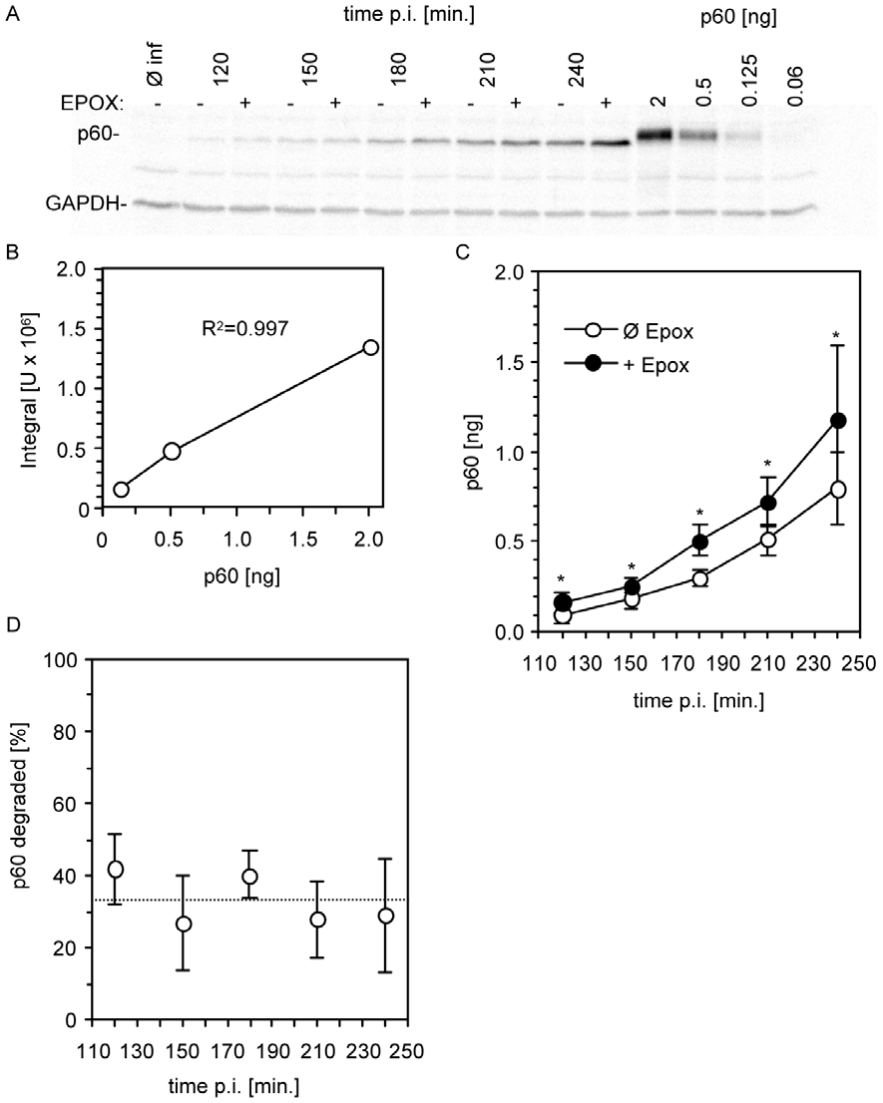
Degradation of p60 in infected cells. Cells were infected with *L. monocytogenes* in the presence (+EPOX) or absence (Ø EPOX) of the proteasome inhibitor epoxomicin. At the indicated time points post infection cells were harvested and intracellular p60 was detected by western blotting. Shown is a representative western blot (A). The absolute amount of p60 was quantified by interpolation with an external p60 standard (B). From the total amount of p60 in the presence and absence of epoxomicin (C) the percentage of degraded p60 was calculated (D). The broken line indicates the average percentage of degraded p60 calculated over all five time points (33%). Shown are the means and standard deviations of four independent experiments. Asterisks (*) indicate a statistical significant (p<0.05) difference between matched values of both experimental groups.

In order to decide between the two antigen processing models described above the possible presence of p60-RDP in infected cells was investigated. In order to detect putative p60-RDP we quantified the percentage of intracellularly degraded p60 in infected cells by western blotting in the presence or absence of the proteasome inhibitor epoxomicin. The absolute amount of p60 in infected cells was determined by quantitative p60 western blot analysis and subsequent interpolation with an external, serially diluted p60 standard. The p60 standard was linear in the range between 0.1 and 2 ng per sample and allowed to measure p60 levels in batches of infected cells between 2 and 4 h post infection (Fig. 2 A–B).

In the presence of epoxomicin compared to untreated controls significantly (p<0.05) more p60 was detected in lysates of *L. monocytogenes*-infected cells between 2 and 4 h post infection (Figure 2A, C). From the amount of p60 in the absence and presence of epoxomicin, the percentage of degraded p60 was calculated (Fig. 2D). The percentage of degraded p60 showed no clear trend over time, therefore an average over all five time points was calculated which was 33% (Fig. 2D).

### Modelling of p60 accumulation in infected cells

**Figure 3:**
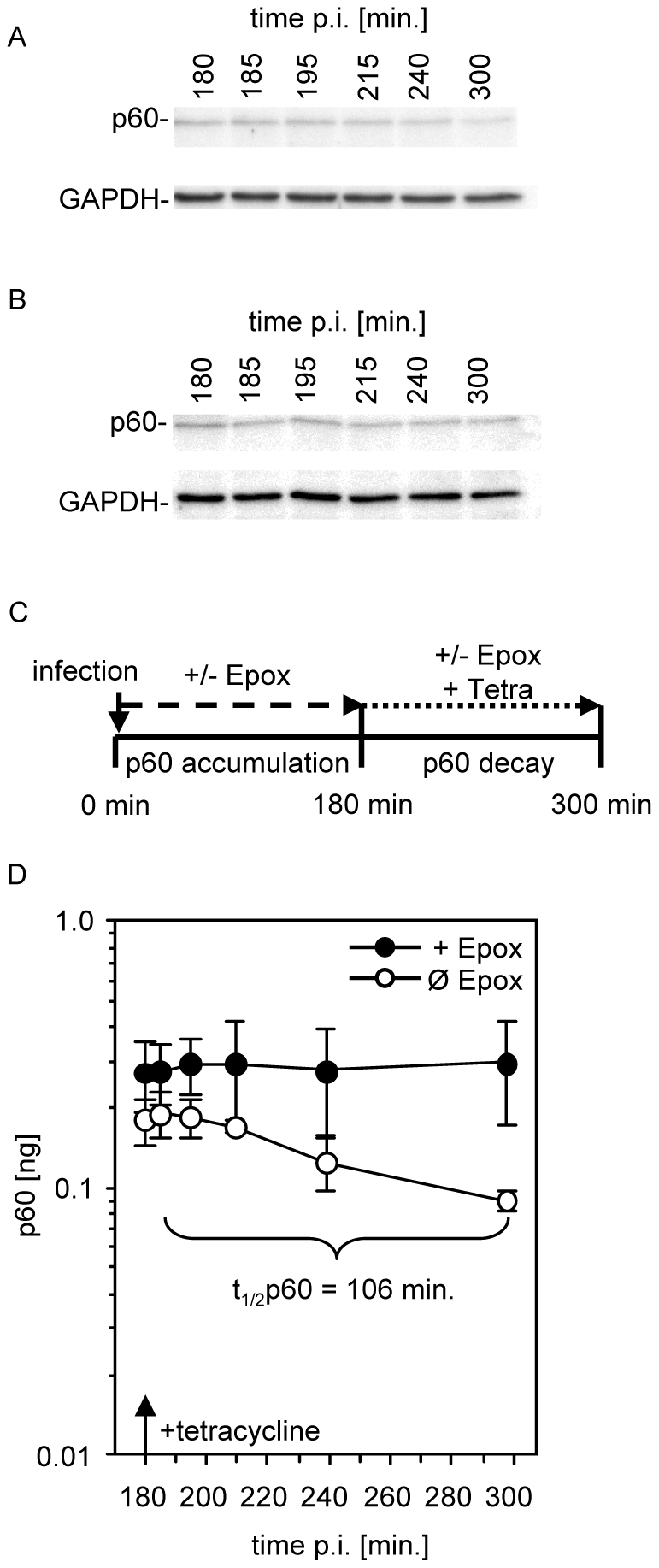
Half-life of p60 in *L. monocytogenes*-infected cells. Three hours after infection further bacterial protein synthesis was inhibited by tetracycline treatment of cells and intracellular p60 was detected by western blot at the indicated time points either in the absence (A) or presence (B) of epoxomicin as shown in the schematic drawing (C). By interpolation with an external p60 standard the total amount of p60 per 100.000 cells was calculated in the presence (+EPOX) or absence (Ø EPOX) of epoxomicin (D). Shown are the means and standard deviations of three independent experiments.

Inhibition of intracellular p60 degradation revealed that between 2 and 4 h post infection approximately 33% of all p60 molecules were degraded intracellularly. Again we used mathematical modelling of p60 antigen processing to decide whether this number is compatible with the hypothesis that putative p60-RDP play a major role as a substrate of the processing of p60-derived antigenic peptides. As prerequisite for these calculations the intracellular half-life of p60 was determined experimentally. For that purpose the decay of p60 was monitored in infected cells after inhibition of bacterial protein-synthesis by addition of tetracycline 3 h post infection (Fig. 3). From the intracellular decay kinetics of p60 the average half-life of intracellularly accumulated p60 was calculated as 106 min (Fig. 3 A and D). No intracellular degradation of p60 occurred in the presence of the proteasome inhibitor epoxomicin (Fig. 3 B and D). At the time point when tetracycline was added, 3 h post infection, significantly (34%, p<0.05) less p60 was recovered from untreated cells compared to epoxomicin-treated cells (Fig. 3 D) thus corroborating the results from figure 2.

**Figure 4:**
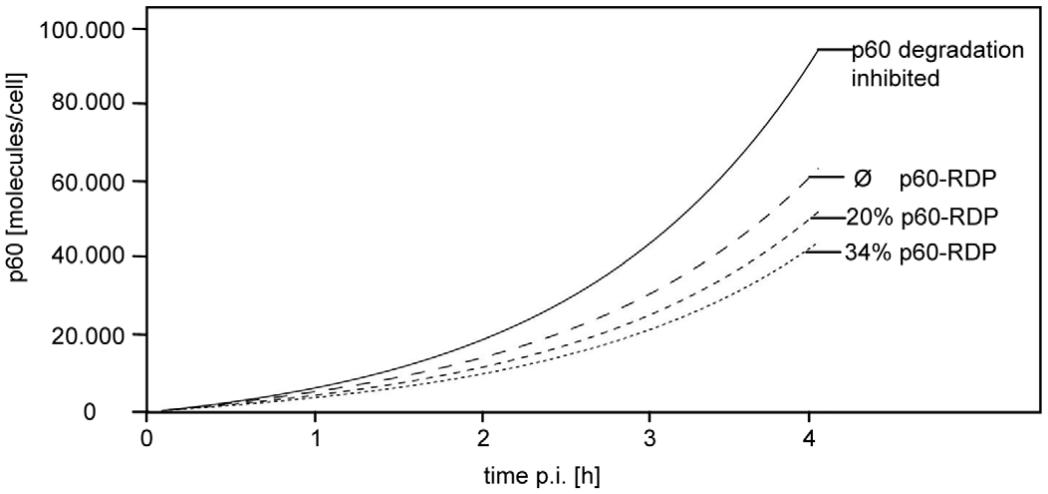
Mathematical modelling of p60 accumulation in infected cells. The intracellular accumulation of p60 was modeled in the absence of p60-RDP using the experimentally determined p60 half-life of 106 min (Ø RDP) or under the assumption that 20% or 31.5% of p60 is rapidly degraded with the predicted half-life of 3.4 min whereas the remaining p60 fraction decays with a half-life of 106 min. In addition the accumulation of p60 was modeled without p60 decay (solid line). For the calculations equation 9 from appendix S1 was used. The initial number o bacteria per cell was 1.25, bacterial doubling time: 60 min, total bacterial p60 synthesis rate 58 molecules/min/bacterium.

Using equation 9 from appendix S1 the accumulation of p60 in infected cells was simulated. In order to estimate the effect of the presence of p60-RDP on the intracellular kinetics of p60 the simulation was performed without p60-RDP using the experimentally determined p60 half-life of 106 min and also with 20% and 31.5% of p60-RDP respectively. For p60-RDP the predicted half-life of 3.4 min was used and for the remaining p60 fraction a half-life of 106 min was accounted. As shown in figure 4 the model clearly predicts that the presence of p60-RDP results in delayed accumulation of intracellular p60 due to a higher percentage of intracellularly degraded p60 molecules.

For the three different scenarios (none, 20%, 31.5% p60-RDP) the percentage of p60 molecules degraded within the first 2, 3 and 4 h after infection of cells was calculated (Table 1). Considering an average p60-RDP rate of 31.5% the model predicts that between 46.1% and 52.1% (average 49.4%) of p60 molecules are degraded, compared to an average of 29% degraded p60 in the absence of RDP. Thus the experimentally determined amount of 33% intracellularly degraded p60 is most precisely predicted by the model without p60-RDP.

**Table 1:** Predicted p60 decay in the presence and absence of p60-RDP.

	p60-RDP^1^		
h post	none	20%	31.5%
infection	predicted p60 decay [%]		
2 h	24.6	38.2	46.1
3 h	29.8	42.6	50.0
4 h	32.7	45.1	52.1
average	29.0	42.0	49.4

1 calculations with RDP were performed on the basis of the predicted p60-RDP half-life of 3.4 min, for the calculation without RDP a p60 half-life of 106 min was used.

We also calculated the absolute number of degraded p60 molecules in the absence and presence of p60-RDP and compared the predicted values with the experimentally determined amount of degraded p60 (Table 2). From the data shown in figure 2, i.e. the amount of p60 and the percentage of decayed p60 molecules the number per cell of decayed p60 molecules was calculated (Table 2). Two, three, and four h after infection, 4678, 14527, and 26854 p60 molecules decayed per cell. The comparison of the experimentally determined numbers with the model calculations shows that the experimentally determined number of decayed p60 molecules per cell correlates most precisely with the predictions from the model without p60-RDP (Table 2). For all time points the predicted values were within the experimentally determined range (mean+/−standard deviation).

**Table 2:** Stoichiometry of p60 in *L. monocytogens*-infected cells.

	decayed p60			
	prediction^1^		experiment^2^	
	p60-RDP			
h post	none	31.5%	mean	SD
infection	[p60 molecules per cell]		[p60 molecules per cell]	
2 h	4626	8675	4678	±879
3 h	13106	21967	14527	±4326
4 h	30851	49090	26854	±22525

1 calculations with RDP were performed on the basis of the predicted p60-RDP half-life of 3.4 min, for the calculation without RDP a p60 half-life of 106 min was used.

2 Means and standard deviations (SD) calculated from 4 experiments.

## Discussion

The current analysis suggests that the experimentally observed tight linkage of bacterial protein synthesis and the presentation of *L. monocytogenes*-derived CD8 T cell epitopes is not caused by the presence of a highly instable antigenic substrate.

This conclusion is based on the assumption that the kinetics of antigen presentation by infected cells depends on the expression kinetics and half-life of the antigenic substrate. Based on this assumption the intracellular decay of p60 was predicted from the kinetics of the presentation of two p60-derived antigenic peptides.

Fitting of a model of antigen presentation that accounts for p60-RDP to experimental data predicts that antigen presentation solely depends on the degradation of a fraction of 31.5% p60-RDP decaying intracellular with an average half-life of 3.4 min while for the remaining p60 a half-life of >300 h was predicted. The very short half-life of p60-RDP elegantly explains the tight connection between p60 synthesis and the presentation of p60-derived antigenic peptides without requiring further assumptions [8], [9].

Obviously, the prediction of an almost inert p60 pool contradicts the experimentally determined intracellular p60 half-life of 90 [12] to 109 min (our data). In regard to antigen presentation, however, the predicted very long half-life has to be interpreted as a p60 pool that does not play a role as substrate for antigen processing and presentation. Because degradation it is not linked to antigen presentation the real intracellular half-life of this pool can not be inferred from the amount of p60-derived antigenic peptides. Thus the interpretation is that the remaining p60 is degraded intracellularly with a half-life of approximately 106 min without generating p60-derived antigenic peptides.

Fitting of the alternative antigen processing model, without p60-RDP predicts that all p60 molecules decay intracellularly with a half-life of 109 min, a prediction which very precisely fits the experimentally measured value of 106 min. This model, however, requires the additional assumption that antigen presentation is somehow linked to bacterial protein synthesis because it does not explain why previously accumulated p60 molecules [13] that after inhibition of bacterial protein synthesis decay with an intracellular half-life of 106 min are not a substrate for the processing of p60-derived antigenic peptides.

Radio-labelling of infected cells for p60 quantification was not used in the current study as the pulsing of cells with radio-labelled amino acids in nutrient-deficient culture medium might shift cells to a catabolic state that influences physiologic cellular protein turnover rates [14]. The p60 half-life determined without radio-labelling (106 min.), however was in the same range as the value of 90 min determined by radio-labelling [12], indicating that this effect had only a minor impact.

In contrast to virus-derived antigenic peptides that are generated with an efficacy of 1 antigenic peptide per several thousand degraded antigens [7], [15] the *L. monocytogenes* p60-derived antigenic peptides p60 217–225 and in p60 449–457 are generated with much higher efficacies of 1 antigenic peptide per 35 and 1 per 1.7 degraded p60 molecules, respectively [11], [12], [13], [16]. Thus in contrast to viral antigenic peptides a very high percentage of an antigen has to be degraded in order to generate sufficient antigenic peptides for antigen presentation.

Both, p60 degradation as well as antigen presentation of p60 217–225 and p60 449–457 are inhibited by proteasome inhibitors [17], [18]. Also after inhibition of bacterial protein synthesis the pool of accumulated p60 molecules is inhibited by proteasome inhibitors, thus linking the degradation of this p60 pool to the proteasome. An important requirement for our experimental approach for the detection of putative p60-RDP is the inhibition of p60 degradation without inhibiting bacterial replication or bacterial protein synthesis. Inhibition of bacterial protein synthesis would result in lower protein levels and such strongly influence the calculation of degraded antigens. We used epoxomicin for all proteasome inhibition experiments because it has the least effect on bacterial replication and p60 synthesis among a panel of various proteasome inhibitors [17].

It is currently unknown, however, why the proteasome-mediated degradation of accumulated p60 molecules does not yield antigenic peptides that are presented in the context of MHC class I molecules [11], [12]. Without the tight linkage to bacterial protein synthesis the presentation of p60-derived antigens should continue after antibiotic inhibition of protein synthesis because a few hours after infection the accumulated p60 molecules could provide antigenic peptides for several hours [13].

It has previously been shown that intracellular *Salmonella* are recognized by the ubiquitin system resulting in spatial association of proteasomes and intracellular bacteria [19], providing a possible direct link between bacteria, bacterial protein translation, and antigen presentation. *Listeria monocytogenes*, however, evades recognition by the ubiquitin system by actin-based motility thus escaping the potential direct association with proteasomes [19]. Triggering of the functional state of the proteasome by the biochemical activity of the bacteria, e.g. by intracellular motility or by protein synthesis, could explain the linkage between antigen processing and de novo protein synthesis. A possible trigger could be provided by viability-associated pathogen-associated molecular patterns such as bacterial messenger RNA which elicits a unique innate response [20]. The association of freshly synthesized unfolded p60 molecules with a cellular chaperone that marks the p60 for later processing by the proteasome [21], [22] or directing of a fraction of newly synthesized p60 to a hypothetical specialized antigen processing compartment [23] seems unlikely as these processes would continue after inhibition of bacterial protein synthesis, which is not supported by the experimental evidence.

Taken together our data strongly suggest that the tight linkage of p60 protein synthesis by *L. monocytogenes* and the processing of p60-derived antigenic peptides is independent of putative p60-RDPs.

## Materials and Methods

### Bacteria and cell lines

All in vitro infections were performed with *L monocytogenes* 43251 [12]. Bacteria were grown at 37°C in brain heart infusion bouillon (BD, Heidelberg, Germany) and used for infection in the logarithmic growth phase. The bacterial concentration was estimated from the OD at 600 nm. The macrophage-like J774A.1 (J774) cell line was purchased from the American Type Culture Collection (Manassas, VA) and was grown in DMEM (Invitrogen, Karlsruhe, Germany) cell growth medium containing 10% FCS (PAA, Pasching, Austria).

### Reagents

The irreversible proteasome inhibitor epoxomicin (SIGMA, Deisenhofen, Germany) was used at concentrations of 2 µM. Epoxomicin was dissolved in dimethyl sulfoxide to yield a 1000× stock solution and stored at −80°C until use. Complete protease inhibitor cocktail tablets were from Roche (Mannheim, Germany) und used according to the suggestions of the manufacturer. DNAse and RNAse were purchased from SIGMA (Deisenhofen, Germany). Tetracyclin (SIGMA) was dissolved in methanol to yield a 500× stock solution (10 mg/ml) stored at −20°C until use.

### Quantitative western blot analysis

J774 cells were grown to 70% confluence in 6 cm cell culture dishes in 4 ml DMEM supplemented with 10% FCS without antibiotics. Cells were infected for 30 min with 0.4 ml of a log phase culture of *L. monocytogens* (OD600 = 0.1). Infected cells were washed twice with DMEM supplemented with 10% FCS and cultured in DMEM with 10% FCS containing 15 µg/ml gentamicin to inhibit extracellular bacterial growth.

Epoxomicin was added 30 min after infection to yield final concentrations of 250 µM and 2 µM, respectively. Tetracycline was added to a final concentration of 20 µg/ml.

At indicated time points cells were washed twice with PBS and lysed in 300 µl of lysis buffer (1× TRIS-buffered saline, 0.1% Triton X-100, Complete protease inhibitor, 50 U/ml DNAse, 50 U/ml RNAse). After 15 min at room temperature lysates were cleared by centrifugation at 14.000 g for 10 min.

Cell lysates were prepared by heating in SDS-PAGE sample buffer (BioRad, München, Germany) for 5 min.

Proteins were separated by SDS-PAGE (10% gel) and blotted onto nitrocellulose (Schleicher & Schuell BioSciences, Dassel, Germany). Blots were blocked in 5% (w/v) dry milk and probed with a 1∶1.000 dilution of a polyclonal rabbit anti-*Listeria moncytogenes p60* antibody (batch R2556) for 4 h and simultaneously probed with rabbit anti-GAPDH and goat anti-GAPDH (abcam, Cambridge, UK) as loading controls in dilutions of 1∶2500 and 1∶1000, respectively. Blots were developed with horseradish peroxidase-conjugated anti-rabbit 1∶5000; DAKOCytomation, Glostrup, Denmark) and enhanced chemiluminescence (Pierce, Rockford, USA). CCD imaging was performed with the LAS-1000 luminescent imaging system (Raytest). Quantification of the amount of p60 of individual samples was performed by interpolation with an external serially diluted p60 standard using AIDA software (Raytest). In order to account for differences in loading volume or protein extraction efficacies all samples were normalized for GAPDH content.

The number of p60 molecules per cell was calculated as molecules_p60_ = m_p60_[ng]/1*10^9^/CE/M_p60_* N_A_, with m_p60_[ng] the total amount in ng of p60 in the sample, CE the number of cell equivalents in the sample, M_p60_ the molar mass of the secreted truncated form of p60 (p60 28–484, M = 4801 g/mol), and N_A_ the Avogadro constant (6.023×10^23^).

### Statistical analysis

The statistical significance of the results was analyzed using the t-test at the 0.05 significance level. Calculations were performed using the WINKS statistical analysis software (TEXASOFT, Cedar Hill, USA).

### Computational simulation of antigen presentation of bacterial antigen synthesis and non-linear regression analysis

In order to model the kinetics of antigen degradation and the formation of peptide/MHC complexes in antigen presenting cells, we used a basic deterministic model [13] (Fig. 1A). Briefly, the model describes the steps involved in antigen processing and presentation by a few well-defined parameters, which can be experimentally determined for a specific bacterial T cell antigen: the bacterial replication rate defined by the bacterial doubling time τ_g_, the bacterial antigen production rate α, the antigen half-life τ_a_, the peptide processing efficacy χ (defined as the ratio of generated peptide/MHC complexes per degraded antigen), and the half-life of the MHC/peptide complex τ_c_. As in other published models [24] the possible limitation of the formation of peptide/MHC complexes by limited supply of nascent MHC class I molecules was not accounted for in order to keep the model as simple as possible [13]. All other peptides which are not protected by complex formation with MHC molecules are rapidly degraded [25].

The previous model was expanded to represent two different states of antigen a_1_ and a_2_, with individual synthesis rates α_1_, α_2_ and individual intracellular half-life τ_a1_, and τ_a2_, respectively. Cells infected with actively replicating bacteria were modeled by a bacterial replication rate >0 and a bacterial protein synthesis rate α = α_1_+α_2_>0. Antibiotic abridgement of infection by treatment with tetracycline was simulated by an infinite bacterial doubling time (τ_g_→∞) and absent bacterial protein synthesis (α_1_ = α_2_ = 0). Cells treated with the proteasome inhibitor epoxomicin were modeled by an infinite antigen half-life (τ_a_→∞). The derivation of the formulas used is described in detail in the supporting information (Appendix S1).

In order to simulate infected cells that were treated with tetracycline after 4 h of active bacterial replication the appropriate equations for t<4 h and t>4 h were merged to yield a stepwise function. This stepwise function was fitted to experimental data by non-linear regression analysis. Regression analysis was performed using NLREG software (www.nlreg.com) running on a personal computer.

## Supporting Information

Appendix S1 Derivation of the formulas used for the modeling of the processing and presentation of bacteria-derived antigenic peptides.
(PDF)

## References

[1] Cresswell P, Ackerman AL, Giodini A, Peaper DR, Wearsch PA (2005). Mechanisms of MHC class I-restricted antigen processing and cross-presentation.. Immunol Rev.

[2] Bienkowski RS, Baum BJ, Crystal RG (1978). Fibroblasts degrade newly synthesised collagen within the cell before secretion.. Nature.

[3] Bienkowski RS, Cowan MJ, McDonald JA, Crystal RG (1978). Degradation of newly synthesized collagen.. J Biol Chem.

[4] Wheatley DN, Giddings MR, Inglis MS (1980). Kinetics of degradation of “short-“ and “long-lived” proteins in cultured mammalian cells.. Cell Biol Int Rep.

[5] Schubert U, Anton LC, Gibbs J, Norbury CC, Yewdell JW (2000). Rapid degradation of a large fraction of newly synthesized proteins by proteasomes.. Nature.

[6] Reits EA, Vos JC, Gromme M, Neefjes J (2000). The major substrates for TAP in vivo are derived from newly synthesized proteins.. Nature.

[7] Princiotta MF, Finzi D, Qian SB, Gibbs J, Schuchmann S (2003). Quantitating protein synthesis, degradation, and endogenous antigen processing.. Immunity.

[8] Qian SB, Reits E, Neefjes J, Deslich JM, Bennink JR (2006). Tight linkage between translation and MHC class I peptide ligand generation implies specialized antigen processing for defective ribosomal products.. J Immunol.

[9] Yewdell JW (2011). DRiPs solidify: progress in understanding endogenous MHC class I antigen processing.. Trends Immunol.

[10] Yewdell JW, Anton LC, Bennink JR (1996). Defective ribosomal products (DRiPs): a major source of antigenic peptides for MHC class I molecules?. J Immunol.

[11] Sijts AJ, Pamer EG (1997). Enhanced intracellular dissociation of major histocompatibility complex class I-associated peptides: a mechanism for optimizing the spectrum of cell surface-presented cytotoxic T lymphocyte epitopes.. J Exp Med.

[12] Villanueva MS, Fischer P, Feen K, Pamer EG (1994). Efficiency of MHC class I antigen processing: a quantitative analysis.. Immunity.

[13] Janda J, Geginat G (2008). A deterministic model for the processing and presentation of bacteria-derived antigenic peptides.. J Theor Biol.

[14] Vabulas RM, Hartl FU (2005). Protein synthesis upon acute nutrient restriction relies on proteasome function.. Science.

[15] Montoya M, Del Val M (1999). Intracellular rate-limiting steps in MHC class I antigen processing.. J Immunol.

[16] Sijts AJ, Neisig A, Neefjes J, Pamer EG (1996). Two Listeria monocytogenes CTL epitopes are processed from the same antigen with different efficiencies.. J Immunol.

[17] Grauling-Halama S, Bahr U, Schenk S, Geginat G (2009). Role of tripeptidyl peptidase II in the processing of Listeria monocytogenes-derived MHC class I-presented antigenic peptides.. Microbes Infect.

[18] Sijts AJ, Villanueva MS, Pamer EG (1996). CTL epitope generation is tightly linked to cellular proteolysis of a Listeria monocytogenes antigen.. J Immunol.

[19] Perrin AJ, Jiang X, Birmingham CL, So NS, Brumell JH (2004). Recognition of bacteria in the cytosol of Mammalian cells by the ubiquitin system.. Curr Biol.

[20] Sander LE, Davis MJ, Boekschoten MV, Amsen D, Dascher CC (2011). Detection of prokaryotic mRNA signifies microbial viability and promotes immunity.. Nature.

[21] Minami R, Hayakawa A, Kagawa H, Yanagi Y, Yokosawa H (2010). BAG-6 is essential for selective elimination of defective proteasomal substrates.. J Cell Biol.

[22] Buchsbaum S, Bercovich B, Ciechanover A (2012). FAT10 is a proteasomal degradation signal that is itself regulated by ubiquitination.. Mol Biol Cell.

[23] Lev A, Princiotta MF, Zanker D, Takeda K, Gibbs JS (2010). Compartmentalized MHC class I antigen processing enhances immunosurveillance by circumventing the law of mass action.. Proc Natl Acad Sci U S A.

[24] Bulik S, Peters B, Holzhutter HG (2005). Quantifying the contribution of defective ribosomal products to antigen production: a model-based computational analysis.. J Immunol.

[25] Reits E, Griekspoor A, Neijssen J, Groothuis T, Jalink K (2003). Peptide diffusion, protection, and degradation in nuclear and cytoplasmic compartments before antigen presentation by MHC class I.. Immunity.

