# Supplementary material for: Linkage of Bacterial Protein Synthesis and Presentation of MHC Class I-Restricted Listeria monocytogenes-Derived Antigenic Peptides
Source: PLoS One. 2012 Mar 12;7(3):e33335. doi: 10.1371/journal.pone.0033335 (PMC3299774; doi:10.1371/journal.pone.0033335)
Supplement: Appendix S1 — Derivation of the formulas used for the modeling of the processing and presentation of bacteria-derived antigenic peptides. (PDF) [file pone.0033335.s001.pdf]

Appendix 1

Modelling of bacterial antigen processing and presentation

In order to model the kinetics of antigen degradation and the formation of peptide/MHC complexes in antigen presenting cells, a basic deterministic model was used [13]. Here this previously reported model is expanded to represent two different states of the same antigen with individual intracellular synthesis rates and half-life.

Model with bacterial replication and protein synthesis

The basic steps involved in antigen presentation were described by a set of linear differential equations which are based on the following basic assumptions:

A bacterium expressing a specific antigen replicates intracellularly with a doubling time τ_g and an intracellular replication rate γ given by:

$$\gamma = \ln 2 / \tau_{\mathrm{g}} \quad (1)$$

Under the assumption that in the first few hours post infection cells are not killed by replicating intracellular bacteria and that after infection the mRNA level of bacteria already is in a steady state or at least rapidly reaches a steady state the bacterial antigen synthesis rate depends only on the number of intracellular bacteria. Then the bacterial growth rate is given by:

$$\dot{g}(t) = \gamma g(t) \quad (2)$$

Intracellular bacteria synthesize an antigen with a defined synthesis rate α, which subsequently is degraded in a first order kinetics with a specific rate β which depends on the half-life τ_a of the antigen:

$$\beta = \ln 2 / \tau_a \quad (3)$$

The rate of antigen synthesis is then given by:

$$\dot{a}(t) = \alpha g(t) - \beta a(t) \quad (4)$$

In order to account for two different states of an antigen, a_1 and a_2, with individual synthesis rates α_1, α_2, intracellular half-life τ_{a1}, and τ_{a2}, and decay rates β_1, and β_2, equation 4 has to be expanded to:

$$\dot{a}(t) = \alpha_1 g(t) - \beta_1 a_1(t) + \alpha_2 g(t) - \beta_2 a_2(t) \tag{5}$$

From the degraded antigen only a peptide-specific fraction χ is correctly processed, and after transport into the endoplasmic reticulum forms peptide/MHC complexes. As in other published models [24] the possible limitation of the formation of peptide/MHC complexes by limited supply of nascent MHC class I molecules was not accounted for in order to keep the model as simple as possible [13]. All other peptides which are not protected by complex formation with MHC molecules are rapidly degraded [25]. The peptide/MHC complexes subsequently decay again with first order kinetics with a specific decay rate δ defined by the half-life τ_c of the peptide/MHC complex:

$$\delta = \ln 2 / \tau_c \tag{6}$$

Thus the rate of formation of an antigen that is present in two different states a_2, and a_2, with individual synthesis rates α_1, α_2, and decay rates β_1, and β_2 is given by:

$$\dot{c}(t) = \beta_1 \chi a_1(t) + \beta_2 \chi a_2(t) - \delta c(t) \tag{7}$$

Solving the system of linear differential equations defined by equations 2, 5, and 7 yields the following terms that describe the time-dependent evolution of the numbers of bacteria g(t), antigen a(t), and peptide/MHC complexes c(t) per antigen presenting cell under conditions of unrestricted bacterial replication. The constant g_0 defines the initial number of microorganisms per cell.

$$g(t) = g_0 e^{\gamma t} \tag{8}$$

$$a(t) = a_1(t) + a_2(t) = \frac{g_0\,\alpha_1}{\beta_1 + \gamma}(e^{\gamma t} - e^{-\beta_1 t}) + \frac{g_0\,\alpha_2}{\beta_2 + \gamma}(e^{\gamma t} - e^{-\beta_2 t}) \tag{9}$$

$$c(t)=\frac{g_0\alpha_1\beta_1\chi}{(\beta_1+\gamma)(\beta_1-\delta)(\gamma+\delta)}(\beta_1(e^{\gamma t}-e^{-\delta t})+\gamma(e^{-\beta_1 t}-e^{-\delta t})+\delta(e^{-\beta_1 t}-e^{\gamma t}))+ \\ +\frac{g_0\alpha_2\beta_2\chi}{(\beta_2+\gamma)(\beta_2-\delta)(\gamma+\delta)}(\beta_2(e^{\gamma t}-e^{-\delta t})+\gamma(e^{-\beta_2 t}-e^{-\delta t})+\delta(e^{-\beta_2 t}-e^{\gamma t})) \quad (10)$$

Model without bacterial replication and protein synthesis

In order to model cells loaded with an antigen that is present in two different states that decay with individual decay rates β_1, and β_2 in the absence of de novo antigen synthesis the basic set of differential equations was solved with $\gamma = 0$ (no intracellular replication) and $\alpha = 0$ (no intracellular antigen synthesis) yielding the following terms for antigen and peptide/MHC complexes per cell:

$$a(t)=a_{01}\,e^{-\beta_1 t}+a_{02}\,e^{-\beta_2 t} \quad (11)$$

$$c(t)=\frac{a_{01}\beta_1\chi}{\beta_1-\delta}(e^{-\delta t}-e^{-\beta_1 t})+\frac{a_{02}\beta_2\chi}{\beta_2-\delta}(e^{-\delta t}-e^{-\beta_2 t}) \quad (12)$$

with a_{01} and a_{02} defining the initial loads with the two different states of antigen.

Equation 12 also describes the generation of new peptide/MHC complexes from accumulated antigen if after an initial period of bacterial replication and protein translation intracellular antigen synthesis is blocked, e.g. by antibiotic treatment of infected cells. Under these conditions the initial antigen loads a_{01} and a_{02} are given by equation 9. Under these conditions, however, also preformed peptide/MHC complexes have to be accounted for. The exponential decay of peptide/MHC complexes generated before addition of the antibiotic is given by:

$$c(t)=c_0 e^{-\delta t} \quad (13)$$

with the initial peptide load c_0 given by equation 10.

Thus the sum of equations 12 and 13 describes the generation and decay of peptide/MHC complexes after antibiotic abridgement of infection:

$$c(t) = \frac{a_{01}\beta_1\chi}{\beta_1 - \delta}(e^{-\delta t} - e^{-\beta_1 t}) + \frac{a_{02}\beta_2\chi}{\beta_2 - \delta}(e^{-\delta t} - e^{-\beta_2 t}) + c_0 e^{-\delta t} \qquad (14)$$

with the initial antigen loads a_{01} and a_{02} given by equation 9 and the initial peptide load c_0 given by equation 10, respectively.

In order to model the kinetics of peptide/MHC complexes in infected cells treated with an antibiotic after an initial period of 4h of unrestricted antigen synthesis a stepwise function combining equations 10 (time period: $0<t<4h$) and 14 (time period: $4h<t<8h$) was used.
